# Quarreling After a Sleepless Night: Preliminary Evidence of the Impact of Sleep Deprivation on Interpersonal Conflict

**DOI:** 10.1007/s42761-021-00076-4

**Published:** 2021-12-07

**Authors:** Patricia Cernadas Curotto, Virginie Sterpenich, David Sander, Nicolas Favez, Ulrike Rimmele, Olga Klimecki

**Affiliations:** 1 Swiss Center for Affective Sciences, University of Geneva, Campus Biotech, 1202 Geneva, Switzerland; 2 Laboratory for the study of Emotion Elicitation and Expression (E3 Lab), Department of Psychology, University of Geneva, Geneva, 1205 Switzerland; 3 Laboratory for Neurology and Imaging of Cognition, Department of Neurosciences, University of Geneva, Campus Biotech, 1202 Geneva, Switzerland; 4 Unité de Psychologie Clinique des Relations Interpersonnelles, Department of Psychology, University of Geneva, Geneva, 1205 Switzerland; 5 Center for the Interdisciplinary Study of Gerontology and Vulnerability, University of Geneva, 1205 Geneva, Switzerland; 6 Emotion and Memory Laboratory, Department of Psychology, University of Geneva, 1205 Geneva, Switzerland; 7 Clinical Psychology and Behavioral Neuroscience, Faculty of Psychology, Technische Universität Dresden, 01187 Dresden, Germany

**Keywords:** Positive affect, Close relationships, Cortisol, Emotions, Couple conflict

## Abstract

Although poor sleep has been found to correlate with deteriorations in romantic relationships, its causal impact on interpersonal conflict has not previously been studied. Therefore, 30 couples were randomly assigned to either a single night of total sleep deprivation or a night of normal sleep to test the effects of sleep deprivation on couples’ conflict. After the experimental night, all participants discussed a topic of recurrent conflict for 15 min. We collected pre- and post-conflict measures of cortisol, self-reports of feelings, and satisfaction with the conflictual discussion. Multilevel analyses revealed higher cortisol levels during conflict and less positive affect prior to and after the conflict for sleep-deprived couples compared to couples in the control condition. These findings provide initial evidence for a causal negative impact of sleep deprivation on couples’ conflicts.

**Supplementary Information:**

The online version contains supplementary material available at 10.1007/s42761-021-00076-4.

## Introduction

During their lifetime, individuals face stressful situations in which the support of their romantic partner may be crucial. Even though a romantic partner may help to buffer external stress (Ditzen et al., 2008), romantic partners can also be the source of tension (Kiecolt-Glaser, 2018). Indeed, marital strain has been reliably linked to higher cortisol levels as well as to other negative consequences for physical health, including immune dysregulation, endocrine changes, and elevations in cardiovascular activity (Kiecolt-Glaser, 2018; Kiecolt-Glaser & Newton, 2001; Miller et al., 1999; Robles & Kiecolt-Glaser, 2003). Even though an extensive body of research has focused on the influence that individual communication styles may have on conflict (Friedlander et al., 2019; Gottman & Notarius, 2002), it is also critical to test the causal influence of external factors. The present study aimed at testing whether sleep deprivation is impacting interpersonal conflict in romantic couples.

In line with this idea, a review has pointed out the role of sleep loss on diverse affective phenomena such as stress and emotions (Ben Simon et al., 2020), which in turn may have an impact on social interactions (Deza-Araujo et al., 2021; Van Kleef, 2009). In particular, sleep deprivation has been shown to increase participant’s self-reported stress (Minkel et al., 2012) and the level of cortisol, which is a bodily response during a stress episode (Leproult et al., 1997; Minkel et al., 2014). Stress seems to have the potential to worsen social relationships: high stress levels can have a detrimental effect on empathic accuracy in women (Crenshaw et al., 2019), and impair cognitive control (Arnsten, 2009), two key factors important for social interactions. Moreover, previous research has revealed associations between self-reported stress and aggression (Hennessy, 2008; Sprague et al., 2011) and between higher stressor induced cortisol levels and punishment behavior (Deza-Araujo et al., 2021).

Likewise, emotions, which can be affected by sleep loss, influence conflicts. First, emotions are inherent to social situations (Van Kleef, 2009), and play a key role in the context of conflicts (Bodtker & Jameson, 2001; Klimecki, 2019). Importantly, the expression of more positive emotions versus negative emotions during interpersonal conflicts has been linked to successful marriages (Driver & Gottman, 2004; Gottman & Levenson, 1992). Second, evidence suggests that emotions are influenced by sleep loss: it has been shown that acute sleep deprivation intensifies negative emotions and reduces positive affect in healthy adults compared to well-rested control participants (Paterson et al., 2011) and in medical residents after several nightshifts (Zohar et al., 2005).

Furthermore, after a total sleep deprivation night, participants showed deficits in emotion recognition (van der Helm et al., 2010), a key process for successful social functioning (Schlegel & Scherer, 2016). In addition, previous research has suggested that sleep deprivation impairs decision-making (Killgore et al., 2006), leads to reduced trust in others, and promotes more aggressive interactions during a social game (Anderson & Dickinson, 2010).

Recent studies found links between shortened sleep or poor sleep quality and more conflictual interactions among romantic partners. Sleepless nights were correlated with more conflict the following day and a night with bad sleep was associated with reduced positive emotions and increased negative emotions during a conflictual discussion in romantic couples as well as a reduced empathic accuracy (Gordon & Chen, 2014). Moreover, couples reporting poor sleep showed greater inflammatory responses as measured by interleukin-6 during a conflict compared to couples who reported a better sleep (Wilson et al., 2017). Self-reported sleep problems have also been shown to correlate with more marital aggression (Keller et al., 2019).

Taken together, there is evidence for a correlation between poor sleep on the one hand and difficulties in social interactions and romantic relationships on the other hand (Gordon & Chen, 2014; Keller et al., 2019; Paterson et al., 2011; Wilson et al., 2017; Zohar et al., 2005). However, the causal link between sleep loss and interpersonal conflict has yet to be determined by testing the impact of sleep deprivation on social interactions (Gordon et al., 2019). Besides, scholars proposed that future studies in this domain will be enriched by combining subjective measures of sleep (e.g., sleep diaries) with objective measures of sleep, such as actigraphy, and by including behavioral measurements of the conflict (Gordon & Chen, 2014; Keller et al., 2019). 

To extend previous findings, the current study aimed at testing the causal impact of one night of sleep deprivation compared to normal sleep on interpersonal conflict in romantic couples. Based on previous studies (Gordon & Chen, 2014; Wilson et al., 2017), we expected that compared to couples with normal sleep, sleep-deprived couples will show more difficulties reaching an agreement, worse emotion recognition, less satisfaction about the conflict discussion, more negative affect and less positive affect, as well as higher cortisol responses during a conflictual discussion.

## Method

### Participants

A total of 30 couples were recruited in Geneva and its surrounding areas through posters and flyers. Posters and flyers indicated that we were recruiting participants for a study on “communication within couples and sleep.” There was no mention of the conflict or sleep deprivation on the posters nor flyers. Both members of each couple completed a demographic questionnaire as well as a series of questionnaires assessing inclusion criteria. Inclusion criteria were the following: no medical, psychiatric, or sleep-related disorder measured by the Pittsburgh Sleep Quality Index (Buysse et al., 1989) and the Epworth Sleepiness Scale (Johns, 1993); no circadian preference assessed by Morningness-Eveningness Questionnaire (Horne & Ostberg, 1976); and no medication, drug consumption, or high energy drinks consumption (more than 5 cups/glasses of coffee/black tea or any other caffeinated drink). As the conflict discussions always took place in the morning, we wanted to ensure that no couple or participant was disadvantaged (e.g., less awake) because of their circadian preference. In addition, couples were recruited if they had been in a relationship between 1 and 5 years. This criterion was adopted as length of relationship can be associated to different ways of managing conflict or different levels of conflict (Whitton et al., 2018). On average, participants had a relationship length of 28.5 months (*SD* = 14.44 months). If both partners met all inclusion criteria, couples were randomly assigned (using a computer-generated list of random numbers) to either a total sleep deprivation condition (*mean age* = 22.9 years, *SD* = 5.01) or a control condition (*mean age* = 21.7 years, *SD* = 1.7). The current study was approved by the Ethics Commission of the Faculty of Psychology and Educational Sciences of the University of Geneva in February 2017. Written informed consent was obtained from every participant. Volunteers received a monetary compensation at the end of the experiment.

### Measures

#### Questionnaires

Sleep hygiene was measured by a series of questionnaires: the Pittsburgh Sleep Quality Index (Buysse et al., 1989), the Epworth Sleepiness Scale (Johns, 1993), and the Morningness-Eveningness Questionnaire (Horne & Ostberg, 1976). Scores on these questionnaires served to control that participants met inclusion criteria (further details on inclusion criteria are provided in the Supplementary Material) and are summarized in Supplementary Table 2. In addition, prior to the first visit to the laboratory, participants were required to complete online questionnaires related to personality traits as well as aspects of their relationship: the Relationship Assessment Scale (Hendrick, 1988), the Commitment in Close Relationship Scale (Bodenmann & Kessler, 2011), the Interpersonal Reactivity Index (Davis, 1983), and the State-Trait Anger Expression Inventory (Spielberger, 2010). These questionnaires were administered to test for potential differences in groups in relationship quality and emotions. Due to an error in sending the online link for these questionnaires to some participants, data from 14 participants are missing on the personality and relationship questionnaires. Thus, the sample size was reduced to 46 participants for the independent *t*-tests and Welch’s *t*-tests (26 individuals in the sleep deprivation and 20 participants in the control condition). These analyses revealed that groups only differed in relationship satisfaction, *t*(44) = 4.04, *p* < 0.001, all other *t*_s_(44) ≤ 0.33 and *p*_s_ ≥ 0.11 (for details, see Supplementary Table 3). Because the relationship satisfaction scores were high (*M* = 4.67, *SD* = 0.32, scale range 1 to 5), we decided to compare them with the scores of the original sample of the Relationship Assessment Scale (Hendrick, 1988) and the ones of the sample used for its French validation (Saramago et al., 2021). Subsequent independent samples *t*-tests indicated that participants in the current study (*N* = 46) were more satisfied with their relationships, *p*_s_ < 0.001, than participants of the Hendrick’s study and participants of the French sample of Samarago et al. (means and standard deviations can be found in the Supplementary Material).

#### Stress Measures

**Fig. 1:**
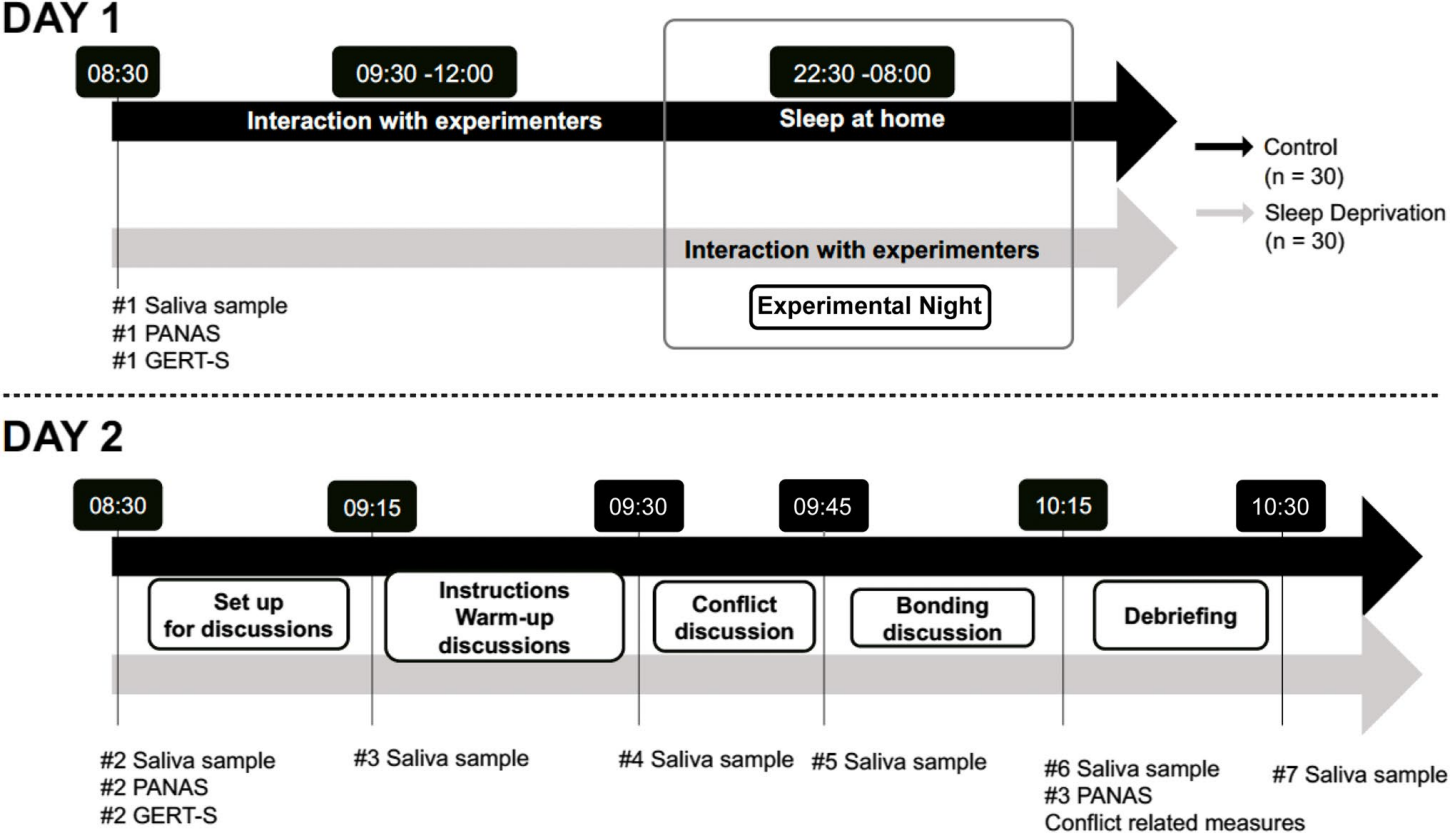
Study procedure for participants in both conditions (sleep deprivation, control condition). PANAS, Positive Affect Negative Affect Schedule; GERT-S, Geneva Emotion Recognition Test

To measure the level of the stress hormone cortisol, saliva samples were collected using Salivette tubes (Salivette, Sarstedt, Nümbrecht, Germany). The first saliva sample was collected on day 1 at 8:30 a.m. and the second one on the following day (day 2) at the same time (8:30 a.m.). The next saliva samples were collected throughout the experiment on day 2 (see Fig. 1). The saliva samples were then stored at − 20 °C and sent to the Clinical Psychology and Psychotherapy Laboratory (University of Zürich) for analysis. Cortisol levels were calculated and expressed in nmol/l.

#### Emotion Recognition

To assess whether sleep deprivation impacts dynamic emotion recognition, which in turn may breed conflict, the short version of the Geneva Emotion Recognition Test (GERT-S; Schlegel & Scherer, 2016) was administered. This task includes 42 short video clips (3 s each) displaying 10 actors who express 14 different emotions (e.g., irritation, pride, and interest). Importantly, the video clips are multimodal: the participants were able to hear actors’ voices and watch facial and body expressions. Clips were presented one by one and we instructed participants to determine after each clip the emotion that was expressed by the actor. Participants could choose among 14 different emotions. Each correct answer was scored 1 and incorrect answer was scored 0; resulting in a total score from 0 (no emotion identified correctly) to 42 (all emotions correctly identified).

#### Emotions Felt

Self-reports of affective states were collected at three time points using the Positive Affect Negative Affect Schedule (PANAS; Watson et al., 1988). This questionnaire comprises 10 items assessing positive affect (e.g., enthusiastic) and 10 items assessing negative affect (e.g., hostile). Participants used a scale from 1 (*not at all*) to 5 (*very much*) to rate each item. In the present study, all participants filled in the PANAS pertaining to their current affect on the morning of day 1, on the morning of day 2, and after the last discussion on day 2.

#### Conflict Discussion and Bonding Discussion

To induce a conflict, we used a well-established paradigm — the Conflict Discussion (Gottman et al., 1977; Levenson & Gottman, 1983, 1985). Each couple was first asked to jointly find and list three topics of recurrent conflict as well as three topics of regular agreement in their relationship (e.g., time spent together, food, housework, and friends). The couple rated jointly how severe each disagreement topic listed was from 0 (*not at all*) to 10 (*totally*). Couples then chose one of the topics of disagreement listed to discuss it for 15 min while trying to resolve it. We used the rating related to the topic that was discussed as the pre-conflict measurement of conflict severity. After the 15 min discussion about the topic of disagreement (conflict discussion), participants switched to the bonding discussion (i.e., discussing about a regular agreement). After the bonding discussion, we asked participants individually to complete a questionnaire using scales from 0 (*not at all*) to 10 (*totally*) to assess (i) conflict severity (post-conflict measurement), (ii) the satisfaction about the agreement (if they reached an agreement after the conflict discussion), and (iii) the satisfaction about the content of the conflict discussion. Participants also indicated whether they succeeded in reaching an agreement during the conflict discussion (yes or no). We asked no questions related to the bonding discussion as the function of this discussion was to calm participants down after the conflict discussion. In addition to these self-reported measures, we also videotaped the conflict discussion and the bonding discussion. Data from these videos may be analyzed by trained coders using coding systems such as the Specific Affect Coding System (Coan & Gottman, 2007).

### Procedure

Participants interested in the study received an online link to the consent form via e-mail. Upon its completion, participants received an online link including a series of questionnaires to assess inclusion criteria (for details, see “Participants” section above). Couples in which both members met all inclusion criteria received a link to a second set of questionnaires to assess personality traits and relationship aspects. After the completion of these questionnaires, participants received sleep diaries and the sleep actigraphs to estimate the duration and quality of their sleep during the four consecutive nights preceding the experiment. Groups did not differ in terms of sleep duration and sleep quality before the experiment in the laboratory, i.e. before day 1 (more details are provided in the Supplementary Material). In addition, participants in both conditions were asked to not drink energy drinks on day 1 (before the experimental night). Before the experimental night, all participants were asked to arrive at 8.30 a.m. at the laboratory to complete the baseline measures (see Fig. 1). Laboratory sessions were grouped: three couples of the same condition were invited jointly. Experimenters first collected saliva samples. Next, participants completed the Positive Affect Negative Affect Schedule (Watson et al., 1988) and the Geneva Emotion Recognition Test (Schlegel & Scherer, 2016). Then, participants in the sleep deprivation condition left the laboratory. Participants in the control condition (three couples each time) stayed for 3 h of interaction with the experimenters to ensure that both groups were familiar with the experimenter and experienced similar situations (for instance, bonding with other participants). Participants in the control group were then asked to spend a normal night of sleep at home, under actigraphy control. Participants in the sleep deprivation condition came back to the laboratory at 10:30 p.m. to complete their sleep deprivation night under the continuous supervision of an experimenter (see Supplementary Material for further details on the sleep deprivation night procedure). The next morning, participants in the sleep deprivation and in the control conditions (after having spent a night of normal sleep at home) were invited for breakfast in the laboratory at 8.00 a.m. Importantly, no caffeinated products were served during the sleep deprivation night or during breakfast. After breakfast, saliva samples were collected at 8.30 a.m., participants completed the Positive Affect Negative Affect Schedule (Watson et al., 1988) and the Geneva Emotion Recognition Test (Schlegel & Scherer, 2016) again. Upon its completion, each couple was invited to sit in a soundproof room. In the room, two cameras, each one facing one participant, were installed to videotape the discussions. Once both members of the couple were seated, a third saliva sample measure was collected. Before the conflict discussion started, participants were instructed to have a 5-min warm-up baseline discussion about the previous week. This discussion served to familiarize participants with the situation in the lab, including the cameras. Then, participants received instructions for the conflict discussion and the bonding discussion: they were asked to come up jointly with three topics for the conflict discussion and three topics for the bonding discussion, and to list them. In addition, for the three topics of disagreement, couples evaluated together the degree of disagreement (conflict severity). Before starting the conflict discussion, saliva samples were collected for the fourth time. After 15 min, the experimenters stopped the discussion, entered in the room, and collected saliva samples again (fifth measure). They then asked couples to switch to the bonding discussion for 15 min. At the end of the allotted time, a sixth saliva sample was taken, and experimenters asked participants to fill in self-report measures about the severity of the conflict, whether they found an agreement, the satisfaction about the agreement, and the satisfaction about the content of the conflict discussion. Participants also completed the Positive Affect Negative Affect Schedule (Watson et al., 1988) for the third time. At the end of the experiment, the aims of the study were revealed to the participants, they were paid for their participation, and the last saliva sample was collected.

### Data Analysis

First, independent *t*-tests on demographical data, sleep-related questionnaires, and personality and relationship questionnaires were conducted to test whether groups differ on any of these measures (details are summarized in Supplementary Tables 2 and 3). Independent *t*-tests indicated that groups only differed significantly on relationship satisfaction. More precisely, couples in the sleep deprivation condition reported lower levels of relationship satisfaction (*M* = 4.52, *SD* = 0.32), compared with couples in the control condition (*M* = 4.86, *SD* = 0.22). To ensure that the differences in relationship satisfaction did not impact the sleep deprivation effect on the dependent variables, scores of relationship satisfaction were included as a covariate in each analysis. To test our hypotheses, data were analyzed using multilevel linear models (MLMs), also known as random effects models or linear mixed models (Fitzmaurice et al., 2011; Hoffman & Rovine, 2007). MLMs were chosen due to their ability to model multiple hierarchical levels of repeated data clustering (time points nested in subjects, and subjects themselves nested in dyads), as well as variables that vary continuously within repeated measures. In addition, MLMs can handle missing data by not requiring that all repeated measures are fully observed at lower levels of the data hierarchy. Missing information in these levels is implicitly imputed using observed information pooled at higher levels, under a Missing at Random (MAR) assumption (Fitzmaurice et al., 2011). As such, missing time-level information does not lead to the removal of an entire subject, and missing subject-level information does not lead to the removal of an entire dyad. The latter was especially important for our covariate adjustment of relationship satisfaction, which was missing in 14 subjects but did not impact the available number of dyads for the final model.

For the cortisol data, self-reported emotions data, and conflict severity data, the first level was accounting for the measurement time (i.e., 7 for cortisol data, 3 for self-reported emotions, and 2 for severity of the conflict). The second level concerned the individual characteristics and the third level the dyads. Regarding satisfaction about the agreement and about the content of the discussion, level 1 concerned individual characteristics and level 2 the dyads.

Modelling with MLMs proceeded in two steps, (1) random effects selection and (2) fixed effects selection. During random effects selection, a model was fitted with fixed effects for the time × condition design, adjusted for relationship satisfaction. Conditional on these effects, two random effects structures were compared for goodness-of-fit, one containing only a random subject intercept, versus one containing a random subject and a random dyad intercept. The structure that minimized the Akaike Information Criterion (AIC) was chosen as the final random effects structure. Following this, we proceeded to the fixed effects selection step, which consisted of a conventional type II ANOVA breakdown of the MLM model with *F*-tests, testing the two-way interaction first (i.e., time × condition), followed by main effects. Relationship satisfaction scores were included as a covariate in each ANOVA. Planned contrasts of group comparisons at specific time points were conducted using *t*-tests within the MLM. As a measure of effect size, we report partial marginal *R*^2^ for *F*-tests, and standardized regression coefficients for *t*-tests.

All analyses were conducted with R version 3.5.1. with the packages “psych” (Revelle, n.d.), “parameters” (Lüdecke et al., 2020), “effectsize” (Ben-Shachar et al., 2020), “r2glmm” (Jaeger, 2017), “lme4” (Bates et al., 2015), and “lmerTest” (Kuznetsova et al., 2017) for multilevel modelling.

## Results

### Sleep Deprivation Increases Cortisol Levels During the Conflict Discussion

To test whether sleep-deprived couples were more stressed by the conflict discussion than couples with normal sleep, multilevel linear models (MLMs) and planned contrasts were conducted. Data analysis of cortisol levels using MLMs indicated that the best-fitting random effects structure was the one accounting for a random subject and random dyad intercept. In other words, there was evidence for within-subject correlation as well as within-dyad correlation of cortisol levels. The model specified in R (using the package lme4) was:

$$\mathrm{lmer}(\mathrm{cortisol}\:\sim\:\mathrm{condition}\;\ast\;\mathrm{time}+\mathrm{relationship}\;\mathrm{satisfaction}+(1\vert\mathrm{id}\;\mathrm{subject})+(1\vert\mathrm{id}\;\mathrm{couples}))$$

**Fig. 2:**
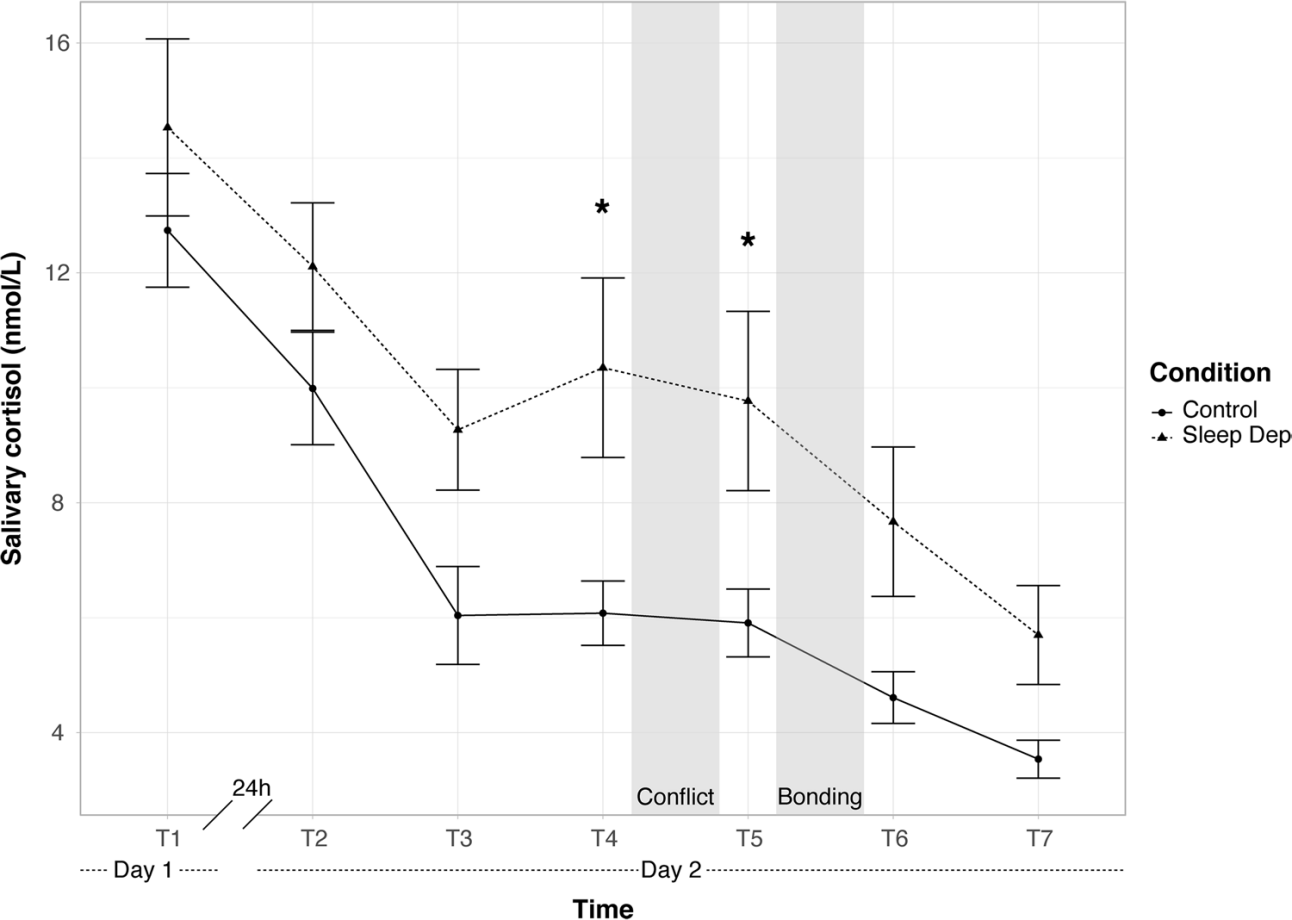
Mean of cortisol levels (nmol/l) as a function of condition (sleep deprivation and control condition). *p < 0.05. Bars represent ± 1 standard errors of the mean. Sleep Dep, sleep deprivation

The time (T1, T2, T3, T4, T5, T6, T7) × condition (control vs sleep deprivation) ANOVA showed no evidence for an interaction of time × condition, *F*(6, 262.144) = 1.05, *p* = 0.40, partial marginal *R*^2^ = 0.007. Three planned contrasts were conducted to test differences between the condition at T1 (baseline), T2 (after the experimental night), and at T5 (after the conflict). As depicted in Fig. 2, a planned contrast did not reveal any group difference at baseline (T1 day 1), *t*(57.27) =  − 0.57, *p* = 0.57, *β*_*z*_ = 0.18, (95% CI [− 0.44, 0.81]). Likewise, a planned contrast did not indicate any difference in salivary cortisol level between rested couples and sleep-deprived couples at T2, after the night of sleep deprivation, *t*(57.27) =  − 0.99, *p* = 0.33, *β*_*z*_ = 0.32, (95% CI [− 0.31, 0.95]). Importantly and in line with our hypotheses, sleep-deprived couples experienced significantly higher cortisol levels during the conflict phase (T5) compared to couples who rested, *t*(57.27) =  − 2.25, *p* = 0.028, *β*_*z*_ = 0.72, (95% CI [0.09, 1.35]). It should be noted that sleep-deprived participants also had higher level of cortisol than rested couples before the conflict (T4), *t*(58.90) =  − 2.29, *p* = 0.026, *β*_*z*_ = 0.74, (95% CI [0.11, 1.37]), an effect size similar to the one at T5. Measures collected at T4 reflect cortisol levels when couples received the conflict discussion instructions and were asked to list a series of disagreement topics as well as agreement topics. Further details of the ANOVA and other analyses at different time points can be found in the Supplementary Material.

### Sleep Deprivation Decreases Positive Affect

Using MLMs and planned contrasts, we then tested whether sleep-deprived couples experienced less positive affect (PANAS) than couples who slept at home. MLMs indicated that the optimal random effects structure was the one including a random subject intercept:

$$\mathrm{lmer}(\mathrm{positive}\;\mathrm{affect}\:\sim\:\mathrm{condition}\;\ast\;\mathrm{time}+\mathrm{relationship}\;\mathrm{satisfaction}+(1\vert\mathrm{id}\;\mathrm{subject}))$$

**Fig. 3:**
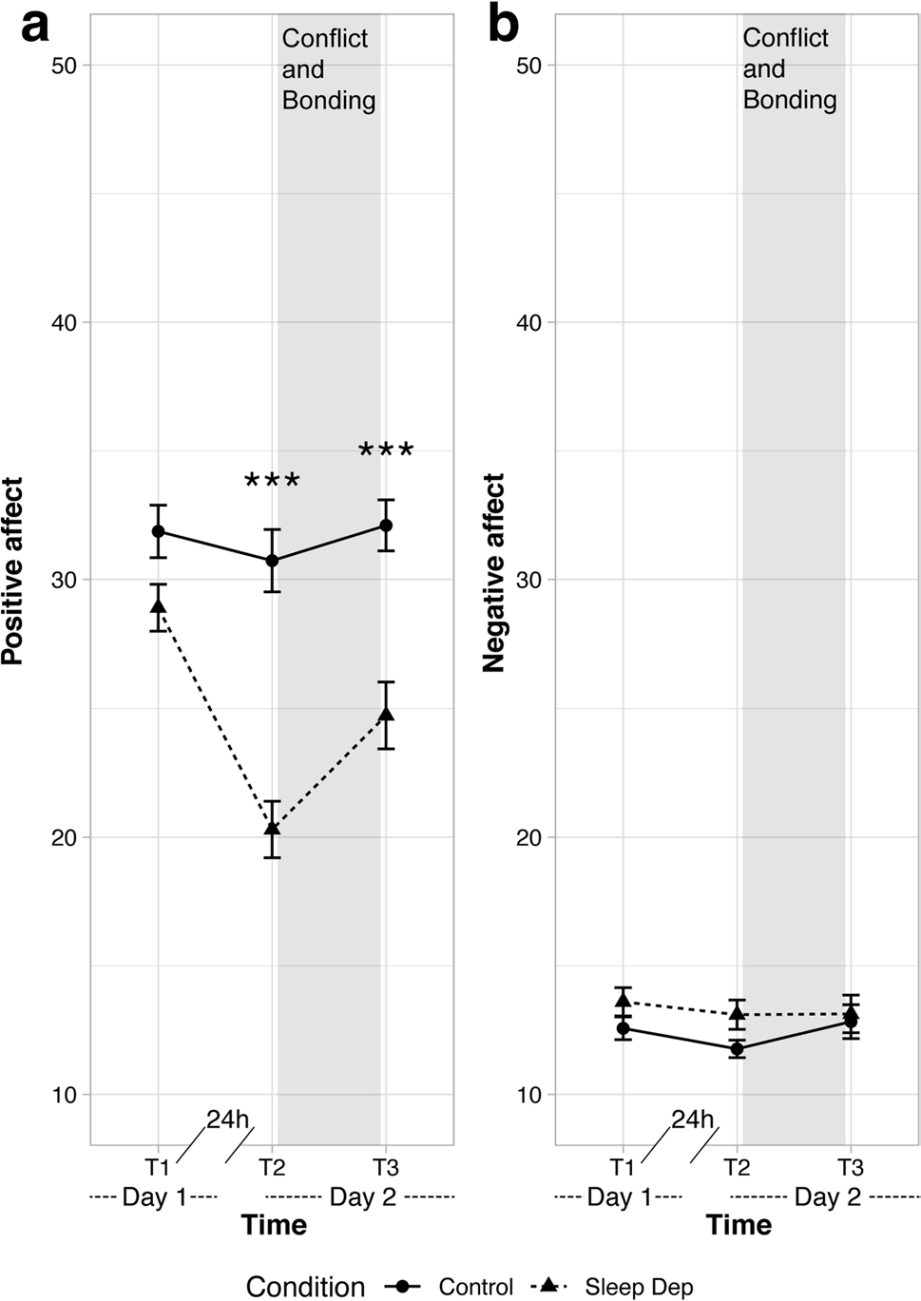
Mean of positive (**a**) and negative (**b**) affect levels (assessed by the Positive Affect Negative Affect Schedule) as a function of condition (sleep deprivation and control condition). ***p < 0.001. Bars represent ± 1 standard errors of the mean. Sleep Dep, sleep deprivation

A time (T1, T2, T3) × condition (control vs sleep deprivation) ANOVA breakdown of fixed effects and planned contrasts were calculated (details are in the Supplementary Material). As shown in Fig. 3a, a planned contrast revealed  no difference for both groups on their self-reported positive affect on day 1 (i.e., T1 prior to the sleep deprivation), *t*(83.65) = 1.39, *p* = 0.17, *β*_*z*_ =  − 0.36, (95% CI [− 0.86, 0.15]). Subsequent planned contrasts revealed that sleep-deprived couples reported less positive affect than control couples at T2 prior to the conflict discussion, *t*(83.65) = 5, *p* < 0.001, *β*_*z*_ =  − 1.29, (95% CI [− 1.79, − 0.78]), and after the conflict discussion at T3, *t*(83.65) = 3.85, *p* < 0.001, *β*_*z*_ =  − 0.99, (95% CI [− 1.5, − 0.49]). To ensure that these significant results were not driven by items related to alertness (i.e., “active,” “alert,” and “excited”), we repeated identical analyses excluding these items. This revealed that the differences between the conditions remained (see Supplementary Material for more details).

Identical to the analyses for positive affect based on all items, MLMs and planned contrasts were conducted on negative affect. Here, MLMs also indicated the same structure:

$$\mathrm{lmer}\;(\mathrm{negative}\;\mathrm{affect}\sim\mathrm{condition}\ast\mathrm{time}+\mathrm{relationship}\;\mathrm{satisfaction}+(1\vert\mathrm{id}\;\mathrm{subject}))$$

As depicted in Fig. 3b, planned contrast confirmed that there were no differences between groups before the experimental manipulation at T1, *t*(88.51) =  − 0.79, *p* = 0.43, *β*_*z*_ = 0.26, (95% CI [− 0.38, 0.91]). Planned contrasts revealed that groups also did not differ in negative affect after the sleep deprivation at T2 and after the conflict discussion at T3 (all *t*_s_(88.51) ≤ 0.11, all *p*_s_ ≥ 0.24; details are in the Supplementary Material).

### Sleep Deprivation Effect on Conflict-Related Measures

The sample size for the subsequent analyses was reduced to 58 participants, due to missing data of one couple in the control condition. We tested whether sleep-deprived participants had more difficulties in finding an agreement during the conflict discussion compared to the participants who slept at home. To this end, a chi-square test was calculated. It revealed no difference between conditions, *p* = 0.63. Indeed, among the 18 couples who reached an agreement at the end of the conflict discussion, 10 were in the sleep deprivation condition while 8 were in the control condition. For those who did not find any agreement, 6 couples were in the control condition and 5 in the sleep deprivation condition.

We compared the satisfaction about the agreement and about the content of the conflict discussion between sleep-deprived and rested couples using MLM analyses. The models included relationship satisfaction scores as a covariate and a random dyad intercept (no repeated measures were done at the level of the subjects):

For satisfaction about the agreement:

$$\mathrm{lmer}(\mathrm{satisfaction}\;\mathrm{agreement}\:\sim\:\mathrm{condition}+\mathrm{relationship}\;\mathrm{satisfaction}+(1\vert\mathrm{id}\;\mathrm{couple}))$$

For satisfaction about the content:

$$\mathrm{lmer}(\mathrm{satisfaction}\;\mathrm{content}\:\sim\:\mathrm{condition}+\mathrm{relationship}\;\mathrm{satisfaction}+(1\vert\mathrm{id}\;\mathrm{couple}))$$

We then used two conventional one-way ANOVAs with the factor condition (control vs sleep deprivation). These analyses did not reveal any main effect of condition on the satisfaction about the agreement and about the content of the conflict discussion (both *p*_s_ ≥ 0.21).

Finally, an MLM analysis and planned contrasts were calculated to measure whether sleep-deprived participants and participants who slept at home differed in their post-conflict ratings of the conflict’s severity. In addition to the covariate (relationship satisfaction scores), the model selected based on the AIC had random subject and dyad intercepts:

$$\mathrm{lmer}(\mathrm{severity}\;\mathrm{conflict}\:\sim\:\mathrm{condition}\;\ast\;\mathrm{time}+\mathrm{relationship}\;\mathrm{satisfaction}+(1\vert\mathrm{id}\;\mathrm{subject})+(1\vert\mathrm{id}\;\mathrm{couples}))$$

A time (T1, T2) × condition (control vs sleep deprivation) ANOVA and planned contrasts did not reveal any difference between sleep-deprived couples and rested couples on their ratings related to the severity of the conflict, at pre-conflict (baseline), *t*(39.10) = 0.67, *p* = 0.50, *β*_*z*_ =  − 0.19, (95% CI [− 0.74, 0.36]), and post-conflict, *t*(38.62) = 1.60, *p* = 0.12, *β*_*z*_ =  − 0.44, (95% CI [− 0.99, 0.01]).

We hypothesized that higher levels of cortisol were linked with higher conflict severity and lower levels of satisfaction about the content and agreement of the conflict discussion. To test whether these relationships between cortisol levels during the conflict (T5) and conflict-related measures existed in the sleep-deprived and control conditions, we ran MLMs. We reported the standardized coefficients. Cortisol levels were treated as an independent variable in the model as they preceded the ratings on conflict-related measures in time. To account for the within-dyad correlation of individuals, we specified each model at the dyad level:

For satisfaction about the agreement:

$$\mathrm{lmer}(\mathrm{satisfaction}\;\mathrm{agreement}\:\sim\:\mathrm{condition}\;\ast\;\mathrm{cortisol}+(1\vert\mathrm{id}\;\mathrm{couple}))$$

For satisfaction about the content:

$$\mathrm{lmer}(\mathrm{satisfaction}\;\mathrm{content}\:\sim\:\mathrm{condition}\;\ast\;\mathrm{cortisol}+(1\vert\mathrm{id}\;\mathrm{couple}))$$

For conflict severity after the conflict discussion:

$$\mathrm{lmer}(\mathrm{post}-\mathrm{conflict}\;\mathrm{severity}\:\sim\:\mathrm{condition}\;\ast\;\mathrm{cortisol}+(1\vert\mathrm{id}\;\mathrm{couple}))$$

MLMs indicated a trend in sleep-deprived couples for a negative relationship between satisfaction about the agreement and cortisol levels at T5, *β*_*z*_ =  − 0.07, *p* = 0.059 (95% CI [− 0.14, 0.00]). No significant relationship was found for the couples in the control condition, *β*_*z*_ = 0.02, *p* = 0.82 (95% CI [− 0.15, 0.18]). Neither the relationship between the ratings of the satisfaction about the conflict’s content and cortisol levels at T5, *β*_*z*_ =  − 0.19, *p* = 0.16 (95% CI [− 0.46, 0.07]), nor the relationship between conflict severity rated after the conflict discussion and cortisol levels at T5, *β*_*z*_ = 0.06, *p* = 0.67 (95% CI [− 0.22, 0.35]), were significant in sleep-deprived couples. None of these relationships among couples in the control condition was significant, all *p*_s_ ≥ 0.43.

### No Sleep Deprivation Effect Was Found on Emotion Recognition

Finally, we tested whether sleep-deprived couples presented lower scores on emotion recognition (assessed by the GERT-S) after a sleepless night compared to couples who slept at home. MLMs indicated that the optimal random effects structure was the one including a random subject intercept and random dyad intercept:

$$\mathrm{lmer}\;(\mathrm{GERT}\;\mathrm{score}\:\sim\:\mathrm{condition}\;\ast\;\mathrm{time}\:+\:\mathrm{relationship}\;\mathrm{satisfaction}\:+\:(1\vert\mathrm{id}\;\mathrm{subject})\:+\:(1\vert\mathrm{id}\;\mathrm{couple}))$$

Consequently, a time (T1, T2) × condition (control vs sleep deprivation) ANOVA and a planned contrast testing whether groups differed on emotion recognition after the experimental night were performed. The planned contrast did not show any difference between sleep-deprived participants and participants who slept at home, *t*(28.56) = 0.70, *p* = 0.49, *β*_*z*_ =  − 0.27, (95% CI [− 1.03, 0.49]).

## Discussion

The current study aimed to test the causal impact of one night of sleep deprivation on interpersonal conflicts in romantic partners. The present findings show increased cortisol levels and less positive emotions related to a conflict discussion in sleep-deprived couples compared to rested couples.

Previous studies have reported both lower and higher levels of cortisol in general after sleep deprivation (Meerlo et al., 2008; Vargas & Lopez-Duran, 2020). The present results did not reveal any difference in cortisol levels between sleep-deprived couples and couples who slept at home after the experimental night. However and importantly, sleep-deprived couples showed higher cortisol levels during the conflict discussion than couples who slept at home. In addition, a trend was found for a negative relationship between cortisol levels during the conflict discussion and satisfaction about the agreement in sleep-deprived couples. These findings dovetail with the observation that elevated cortisol levels by an external stressor worsen social interactions (Deza-Araujo et al., 2021), and with research showing that high self-reported stress is associated to aggression (Hennessy, 2008; Sprague et al., 2011). The current results are consistent with previous research showing that stress reactivity is more elevated (i.e., higher cortisol levels) in sleep-deprived individuals compared to control participants when subsequently exposed to a stressor (Minkel et al., 2014). In this sense, conflict itself could be considered a stressor in the present study, and sleep-deprived couples may have shown amplified reactivity compared to rested couples. However, it should be noted that another study found that individuals did not show higher stress reactivity after a single sleep deprivation night compared to well-rested individuals (Schwarz et al., 2018). Overall, these conflicting results suggest that relationships between sleep deprivation and cortisol levels are complex and that different types of sleep deprivation manipulations and stressors produce different cortisol levels (Schwarz et al., 2018). Future studies with larger sample sizes are needed to shed more light on this issue.

With regard to self-reported emotions, sleep-deprived couples reported fewer positive emotions compared to rested couples, providing further support to the growing body of research establishing a link between sleep loss and a reduction in positive affect (Finan et al., 2017; Zohar et al., 2005). In the present study, sleep-deprived participants also indicated less positive feelings after the conflict discussion compared to participants in the control condition. This is consistent with previous findings linking self-reported poor sleep with reduced positive emotions observed by coders in relationship conflicts (Gordon & Chen, 2014). The emotional alterations found in the present study (i.e., increased cortisol and decreased positive affect) could be related to an overactivation of the amygdala and a decreased functional connectivity with the prefrontal cortex, a phenomenon that has been described after a total sleep deprivation (Yoo et al., 2007).

Regarding negative feelings, previous evidence points to increased negative affect after one night of sleep deprivation (Yoo et al., 2007). Furthermore, previous correlational studies suggest that poor sleep was associated to less conflict resolution, reduced emotion recognition, and increased aggression (Gordon & Chen, 2014; Keller et al., 2019; van der Helm et al., 2010). The present study did not corroborate any of these findings. This might be due to (i) the relatively small sample size, (ii) the relatively short conflict discussion, (iii) the timing of data collection for conflict measures (i.e., after the bonding discussion), (iv) the use of self-reports to assess negative feelings, and (v) the overrepresentation of satisfied couples in our research, which is a common issue in the field (Wilson et al., 2017). An additional potential confound is that participants in the control group slept at home and may differ from sleep-deprived participants by not being together during the experimental night. Moreover, the interpersonal interactions during the sleep deprivation may have buffered sleep loss effects by eliciting and influencing participants’ emotions (Van Kleef, 2009). Finally, although our study addresses causality, the sleep deprivation procedure lacks ecological validity. Major reasons for sleep loss in couples most likely include working night shifts or having young children. Therefore, future research should test sleep loss over longer time spans or repeated awakenings.

The present results are only a first step in providing causal evidence for the impact of sleep deprivation on couple conflict. Future studies with larger sample sizes are needed to replicate these results and to explore the role of cortisol as a biological mediator of situational stressors (including sleep deprivation) on conflict processes in more depth. Furthermore, studies on couple conflict could also adopt paradigms in which the conflict discussion is longer (e.g., 60 min) and complement self-reports by including more biological measures, such as functional magnetic resonance imaging (Rafi et al., 2020). Additionally, further work should adopt strategies to recruit dissatisfied couples to measure the impact of sleep loss in unhappy couples. Indeed, it remains unanswered whether the negative impact of sleep loss on relationship conflict found here would be more severe in less satisfied couples. In addition, there is a need to explore psychological mechanisms underlying the adverse effects of sleep loss on social interactions. In line with this idea, scholars have started to explore many processes such as impaired empathic accuracy (Gordon & Chen, 2014), attentional biases (Finan et al., 2017; Nota & Coles, 2018), reduced ability to regulate one’s own emotion (Mauss et al., 2013), or lower self-control (Keller et al., 2019). It should be noted that the current study did not find an effect of sleep loss on emotion recognition. This is consistent, however, with previous research showing that one night of sleep deprivation was not associated with decreased emotion recognition when using multimodal stimuli (Holding et al., 2017).

The current findings align with a review establishing that an appropriate sleep (duration and quality) is crucial for having an adaptive social and emotional functioning (Ben Simon et al., 2020). In the future, more causal studies using randomized controlled trials should be carried out in order to replicate these results with larger samples. Additionally, our results may extend to other interpersonal interactions such as the ones happening at the workplace. For instance, it remains unknown whether highlighting the importance of a good sleep hygiene prior to negotiation may favor successful conflict resolution. Encouraging evidence has been already reported in the context of an intervention aiming to reduce insomnia and showing its beneficial effects on work-related outcomes, such as showing concern towards coworkers (Barnes et al., 2017). Consequently, there is an urge to bring together disciplines (sleep research, affective sciences, and social psychology) to account for the effects of sleep loss, to delineate the role of sleep, and, finally, to contribute to a better understanding of social and affective processes (Gordon et al., 2017).

## Supplementary Information

Below is the link to the electronic supplementary material.

Supplementary file1 (DOCX 11.2 MB)

## Additional Information

### Data Availability

Data are publicly available at https://osf.io/dgrj3/?view_only=b50343d0f84246b1a91aad517b403a95.
