# Supplementary material for: Quarreling After a Sleepless Night: Preliminary Evidence of the Impact of Sleep Deprivation on Interpersonal Conflict
Source: Affect Sci. 2021 Dec 7;3(2):341–52. doi: 10.1007/s42761-021-00076-4 (PMC9249692; doi:10.1007/s42761-021-00076-4)
Supplement: Supplementary file 1 — Supplementary file1 (DOCX 11.2 MB) [file 42761_2021_76_MOESM1_ESM.docx]

**Quarreling after a sleepless night: preliminary evidence of the impact of sleep deprivation on interpersonal conflict**

**Authors:** Patricia Cernadas Curotto^1^, Virginie Sterpenich^1,2^, David Sander^1^, Nicolas Favez^3^, Ulrike Rimmele^2,4^, and Olga Klimecki^1,5^

**Affiliations:**

^1^Swiss Center for Affective Sciences, University of Geneva, Campus Biotech, 1202 Geneva, Switzerland

^2^Laboratory for Neurology and Imaging of Cognition, Department of Neurosciences, University of Geneva, Campus Biotech, 1202 Geneva, Switzerland

^3^Unité de Psychologie Clinique des Relations Interpersonnelles, Department of Psychology, University of Geneva, 1205 Geneva, Switzerland

^4^Center for the Interdisciplinary Study of Gerontology and Vulnerability, University of Geneva, 1205 Geneva, Switzerland

^5^Clinical Psychology and Behavioral Neuroscience, Faculty of Psychology, Technische Universität Dresden, 01187, Dresden, Germany

**Couples were highly satisfied about their relationship**

Due to an error, 14 participants were missing for the personality traits and relationships aspects questionnaires (the Relationship Assessment Scale, the Commitment in Close Relationship Scale, the Interpersonal Reactivity Index, and the State-Trait Anger Expression Inventory). As a result, the sample size was reduced to 46 participants: 26 individuals in the sleep deprivation and 20 participants in the control condition.

| **Supplementary Table 1: COMPARISON OF SAMPLES ON RELATIONSHIP SATISFACTION** | | | | | | |
| --- | --- | --- | --- | --- | --- | --- |
| *Independent samples t-tests on the scores of the Relationship Assessment Scale indicated significant differences between the current sample and the original sample of the Relationship Assessment Scale (Hendrick, 1988) and the French validation sample (Saramago et al., 2021).* | | | | | | |
| Relationship satisfaction scores | | | | | | |
|  | *N* | *M* | *SD* | *df* | *t* | *p* |
| Hendrick (1988), study 1 | 125 | 4.16 | 0.92 | 169 | 3.67 | <.001*** |
| Saramago et al. (2021), study 1 | 200 | 4.23 | 0.84 | 244 | 3.49 | <.001*** |
| *Note*. SD = Standard Deviation; df = degrees of freedom.  *** *p* <.001 | | | | | | |

**Inclusion criteria related to sleep-related disorders**

Pittsburgh Sleep Quality Index (PSQI; Buysse, Reynolds, Monk, Berman, & Kupfer, 1989) and the Epworth Sleepiness Scale’s (Johns, 1993) scores were used to check that couples participating in the current study did not present any sleep-related disorders. More precisely, if both partners had scores higher than 5 at the PSQI, which indicates severe difficulties in at least two components assessed by the PSQI (e.g., sleep latency, sleep duration, or use of sleeping medication) or moderate difficulties in more than three components the couple was excluded. In addition, individuals with scores higher than 7 at PSQI were excluded. Both groups did not differ in PSQI scores as depicted in the Supplementary Table 2. Regarding the Epworth Sleepiness Scale, participants showing a score exceeding 15 (Severe Excessive Daytime Sleepiness) for the week or the weekend were excluded.

| **Supplementary Table 2: DEMOGRAPHICAL AND SLEEP HYGIENE MEASURES FOR BOTH GROUPS**  *Independent t-tests indicated no differences as a function of condition (Sleep Deprivation, Control) in demographical measures and baseline measures related to sleep. Variances were not equal for the variable age, thus Welch’s t-test was used here.* | | | | | | | |
| --- | --- | --- | --- | --- | --- | --- | --- |
|  | **Sleep Deprivation**  (n = 30) | | **Control**  (n = 30) | | **Group Difference** | | |
|  |  |  |  |  |  |  |  |
|  | *Mean* | *SD* | *Mean* | *SD* | *t* | *df* | *p* |
| **Age** | 22.9 | 5.01 | 21.7 | 1.7 | -1.24 | 35.64 | .22 |
| **Relationship Length (in months)** | 28.67 | 16.77 | 28.33 | 11.95 | -0.09 | 58 | .93 |
| **Epworth Sleepiness Scale (Week)** | 3.77 | 2.21 | 4.47 | 2.93 | 1.04 | 58 | .30 |
| **Epworth Sleepiness Scale (Weekend)** | 6.77 | 3.43 | 6.8 | 2.92 | 0.04 | 58 | .97 |
| **Pittsburgh Sleep Quality Index** | 3.57 | 1.7 | 3.5 | 1.61 | -0.16 | 58 | .88 |
| **Morningness-Eveningness questionnaire** | 52.15 | 9.02 | 54.9 | 7.99 | 1.25 | 58 | .22 |
| *Note*. SD = Standard Deviation; df = degrees of freedom. | | | | | | | |

**Supplementary Table 3: PERSONALITY AND RELATIONSHIP MEASURES FOR BOTH GROUPS**

*Independent t-tests were used to test for differences as a function of condition (Sleep Deprivation, Control) in relationship as well as traits questionnaires. Variances were not equal for the personal distress scale of the Interpersonal Reactivity Index, thus Welch’s t-test was used here. The only significant difference that emerged between groups was relationship satisfaction, which was lower in the sleep deprivation group. Questionnaires from 14 participants were missing here.*

|  | **Sleep Deprivation**  (n = 26) | | | **Control**  (n = 20) | | **Group Difference** | | |
| --- | --- | --- | --- | --- | --- | --- | --- | --- |
|  |  |  |  |  |  |  |  |  |
|  | | *Mean* | *SD* | *Mean* | *SD* | *t* | *df* | *p* |
| **Relationship satisfaction** | | 4.52 | 0.32 | 4.86 | 0.22 | 4.04 | 44 | <.001*** |
| **Commitment** | | 5.43 | 0.57 | 5.53 | 0.46 | 0.65 | 44 | .52 |
| **Interpersonal Reactivity Index** Perspective-Taking | | 33.62 | 5.19 | 32.75 | 3.92 | -0.62 | 44 | .54 |
| **Interpersonal Reactivity Index** Empathic Concern | | 33.73 | 5.78 | 35.85 | 3.96 | 1.40 | 44 | .17 |
| **Interpersonal Reactivity Index** Fantasy | | 35.5 | 7.61 | 32.75 | 7.6 | -1.22 | 44 | .23 |
| **Interpersonal Reactivity Index** Personal Distress | | 23.12 | 8.28 | 23.75 | 4.7 | 0.33 | 40.89 | .74 |
| **Anger Expression (STAXI)** | | 43.62 | 11.24 | 38.75 | 7.79 | -1.65 | 44 | .11 |
| *Note*. SD = Standard Deviation; df = degrees of freedom; STAXI = State-Trait Anger Expression Inventory.  *** *p*<.001 | | | | | | | | |

| **Sleep duration, sleep quality, and sleep efficiency of the four nights preceding the experiment**  To test whether groups differed on sleep duration and sleep quality, sleep diaries and wrist actigraphy were collected during the four nights preceding the experiment. Sleep diaries required participants to estimate each morning how many hours they had slept (sleep duration) as well as how well they slept (sleep quality) on a scale of 1 (*worst sleep ever*) to 10 (*best sleep ever*). Supplementary Table 4 provides means, standard deviations, and independent *t*-tests for each condition (sleep deprivation vs control) regarding sleep diary ratings (sleep duration and sleep quality) on Night-1 (i.e., the night before the baseline measures were collected), Experimental night (i.e., sleep deprivation night/night of sleep at home), and the average of the four consecutive nights (Night-4, Night-3, Night-2, and Night-1) preceding the experiment. |
| --- |

| **Supplementary Table 4: SLEEP DIARY RATINGS FOR BOTH GROUPS** | | | | | | | |
| --- | --- | --- | --- | --- | --- | --- | --- |
| *Means, standard deviations, independent t- tests of sleep duration and sleep quality for each group on Night-1, on experimental night, and on average of the four consecutive nights (Night-4, Night-3, Night-2, and Night-1) preceding the experiment* | | | | | | | |
| Condition |  |  |  |  |  |  |  |
|  | Sleep deprivation | | Control | |  |  |  |
|  | n = 29 | | n = 30 | |  |  |  |
|  | *M* | *SD* | *M* | *SD* | *t* | *df* | *p* |
| Sleep duration |  |  |  |  |  |  |  |
| Night -1 | 7.02 | 0.9 | 6.84 | 1 | -0.74 | 58 | .46 |
| Experimental night | - | - | 7.31 | 0.92 | - | - | - |
| Average 4 nights | 7.74 | 0.65 | 7.68 | 0.61 | -0.32 | 58 | .75 |
| Sleep quality |  |  |  |  |  |  |  |
| Night - 1 | 7.23 | 1.65 | 7.13 | 1.38 | -0.25 | 58 | .80 |
| Experimental night | - | - | 7.5 | 1.53 | - | - | - |
| Average 4 nights | 7.33 | 1.21 | 6.98 | 1.05 | -1.18 | 58 | .24 |
| *Note*. SD = Standard Deviation; df = degrees of freedom. | | | | | | | |

To collect objective measures of sleep, participants wore an Actimeter GT3X+ (Actigraph, Pensacola, FL) on their nondominant wrist, nonstop, for the four consecutive days and nights that preceded the experiment. As it was not possible to record the data from one of the Actimeters, the sample size for the objective measure of sleep was reduced to 59 participants. We used data reported in the sleep diaries to determine for each night the time when participants went to bed (sleep onset) and got up (wake onset) and included this information in the data recorded by the Actimeters. We then obtained a measure of sleep efficiency in percentage (i.e., division between the total number of sleep minutes by the total number of minutes that the participant was in bed). Using an independent *t*-test, we evaluated whether the groups differed in the total number of sleep minutes and in their score of sleep efficiency of the four nights that preceded the experiment. Independent *t*-test revealed that sleep efficiency scores of participants in the sleep deprivation condition (*M* = 83%, *SD* = 7%) did not differ from the scores of participants in the control condition (*M* = 80%, *SD* = 9%), *t*(57) = -1.51, *p* = .14.

**Sleep deprivation night procedure**


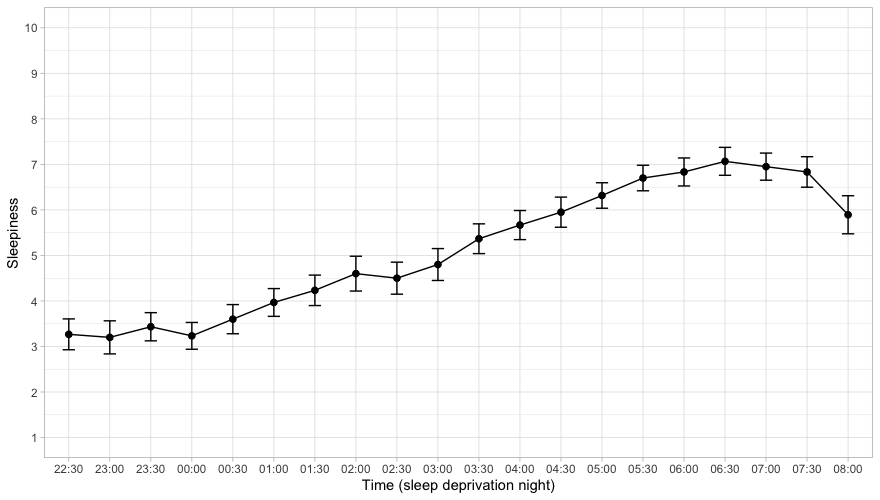
Participants in the sleep deprivation condition were instructed not to nap in the period after the baseline measurements on day 1 and in the period before the sleep deprivation night. Both members of the couples in the sleep deprivation condition were invited to the laboratory at 10:30 pm the evening before the conflict discussion. For each sleep deprivation night, three couples were asked to stay awake together under the constant supervision of an experimenter at the lab. Participants could drink (soft drinks and decaffeinated beverages) and eat at any time during the sleep deprivation night as long as they recorded their consumption on a slip of paper. To keep participants awake, experimenters engaged them in social activities such as playing board games or watching movies. Each half of hour, participants were required to assess their level of sleepiness using the Karolinska Sleepiness Scale (Shahid, Wilkinson, Marcu, & Shapiro, 2011). Participants reported their psycho-physical sate using a 9-point scale ranging from 1 (*extremely alert*) to 10 (*extremely sleepy, falls asleep all the time*). Supplementary Fig. 1 shows sleepiness ratings throughout the sleep deprivation night. Around 8 a.m. participants were invited to have a breakfast before starting the testing period at 8.30 a.m.

**Supplementary Fig. 1** Sleepiness ratings assessed by the Karolinska Sleepiness Scale each half hour among sleep-deprived participants (*n* = 30). Bars represent ±1 standard errors of the mean.

**Results section**

*Cortisol levels during the experiment*

Based on the data analysis of the cortisol levels using multilevel linear models (MLMs), the best-fitting random effects structure was the one accounting for a random subject and random dyad intercept. Conditional on the chosen random effects structure, a time (T1, T2, T3, T4, T5, T6, T7) × condition (control vs sleep deprivation) ANOVA breakdown of fixed effects revealed the following results. There was no evidence for an interaction of time × condition, *F*(6, 262.144) = 1.05, *p* = .40, partial marginal *R*^2^ = .007. A significant main effect of time was found, *F*(6, 262.155) = 16.65, *p* <.001, partial marginal *R*^2^ = .14, while the main effect of condition showed only a trend toward significance, *F*(1, 26.25) = 3.56, *p* =.07, *β_z_* = 0.50, (95% CI [-0.02, 1.01]). Planned contrasts to test whether cortisol levels of sleep-deprived couples differ from couples who slept at home at baseline (T1), after the sleep deprivation (T2) as well as during the conflictual discussion (T5) are reported in the main manuscript. We explored other differences between conditions at T3 (after setting up participants for the discussions), T4 (after having received conflict discussion instructions and while deciding which disagreement and agreement topics to discuss), T6 (after the bonding discussion) and T7 (after the debriefing). A *t*-test revealed that sleep-deprived couples did not differ relative to their rested counterparts at T3, *t*(57.27) = -1.69, *p* = .10, *β_z_* = 0.54, (95% CI [-0.08.,1.17]). As mentioned in the main manuscript, sleep-deprived couples had significantly higher levels of cortisol at T4 than rested couples, *t*(58.90) = -2.29, *p* = .026, *β_z_* = 0.74, (95% CI [0.11, 1.37]). We found no differences between the groups at T6, *t*(57.27) = -1.73, *p* = .09, *β_z_* = 0.56, (95% CI [-0.07, 1.19]), and at T7, *t*(57.27) = -1.29, *p* = .20, *β_z_* = 0.42, (95% CI [-0.21, 1.04]).

As couples in the control condition were not asked to wake up at a certain time, we tested whether wake-up time had an effect on cortisol levels. We first calculated a score in control participants by subtracting the exact wake-up time of each subject from the hour in which we collected the second saliva sample (i.e., 8:30 a.m. on day 2, at T2).We then conducted MLMs among the control couples including this score in hours as a continuous covariable. MLMs indicated that the ideal random effects structure was including a random subject and random dyad intercept:

lmer(cortisol~ time+time awakening+relationship satisfaction+(1|id subject) +(1|id couples))

While the subsequent ANOVA revealed a main effect of time, *F*(6, 114) = 21.23, *p* < .001, partial marginal *R^2^* = .37, no effect of the covariate wake-up time was found, *F*(1, 16.52) = 1.66, *p* = .22.

*Self-reported affect assessed with the Positive Affect Negative Affect Schedule (PANAS)*

Data analyses on positive and negative emotions using MLMs indicated that the optimal random effects structure was the

one including a random subject intercept. Two time (T1, T2, T3) × condition (control vs sleep deprivation) ANOVAs for self-reported positive affect and for negative affect were conducted. Regarding the 3*2 ANOVA on self-reported positive emotions, it yielded a significant main effect of the condition, *F*(1, 43) = 17.07, *p* < .001, *β_z_* = 0.88, (95% CI [ -1.3, -0.46]) and a significant main effect of time, *F*(2, 88) = 18.26, *p* < .001, partial marginal *R^2^* = .13. Moreover, a significant interaction effect (time × condition) was found, *F*(2, 88) = 7.12, *p* = .001, partial marginal *R^2^* = .04.

Additionally, we tested whether the differences found between sleep-deprived participants and control participants with respect to positive affect were not driven by three items of the PANAS related to “alertness” (i.e, “active”, “alert”, and “excited”). To this purpose, we used MLMs analyses on the sum of self-reported positive emotions while excluding these items. Similar to prior analyses on the PANAS, MLMs indicated that the optimal random effects structure was including a random subject intercept:

lmer(positive affect~condition*time+relationship satisfaction+(1|id subject))

A 3*2 ANOVA revealed the same pattern of results for the self-reported positive affect based on all items. A significant main effect of condition, *F*(1, 43) = 11.63, *p* = .001, *β_z_* = -0.74, (95% CI [ -1.17, -0.32]), a significant main effect of time, *F*(2, 88) = 13, *p* < .001, partial marginal *R^2^* = .23, and a significant interaction effect (time × condition) were found, *F*(2, 88) = 3.89, *p* = .024, partial marginal *R^2^* = .15. As depicted in Supplementary Fig. 2, contrasts revealed that groups did not differ on positive affect excluding alertness items on day 1 at T1 (baseline), *t*(87.71) = 1.22, *p* = .22, *β_z_* = -0.33 (95% CI [ -0.85, 0.20]). However, sleep-deprived couples felt less positive affect than couples in the control condition on day 2 at T2 (after the sleep deprivation night), *t*(87.71) = 3.98, *p* <.001, *β_z_* = -1.07 (95% CI [ -1.60, -0.54]), and at T3 (after the conflict discussion), *t*(87.71) = 3.09, *p* = .003, *β_z_* = -0.83 (95% CI [ -1.36, -0.30]), even when alertness items were excluded.


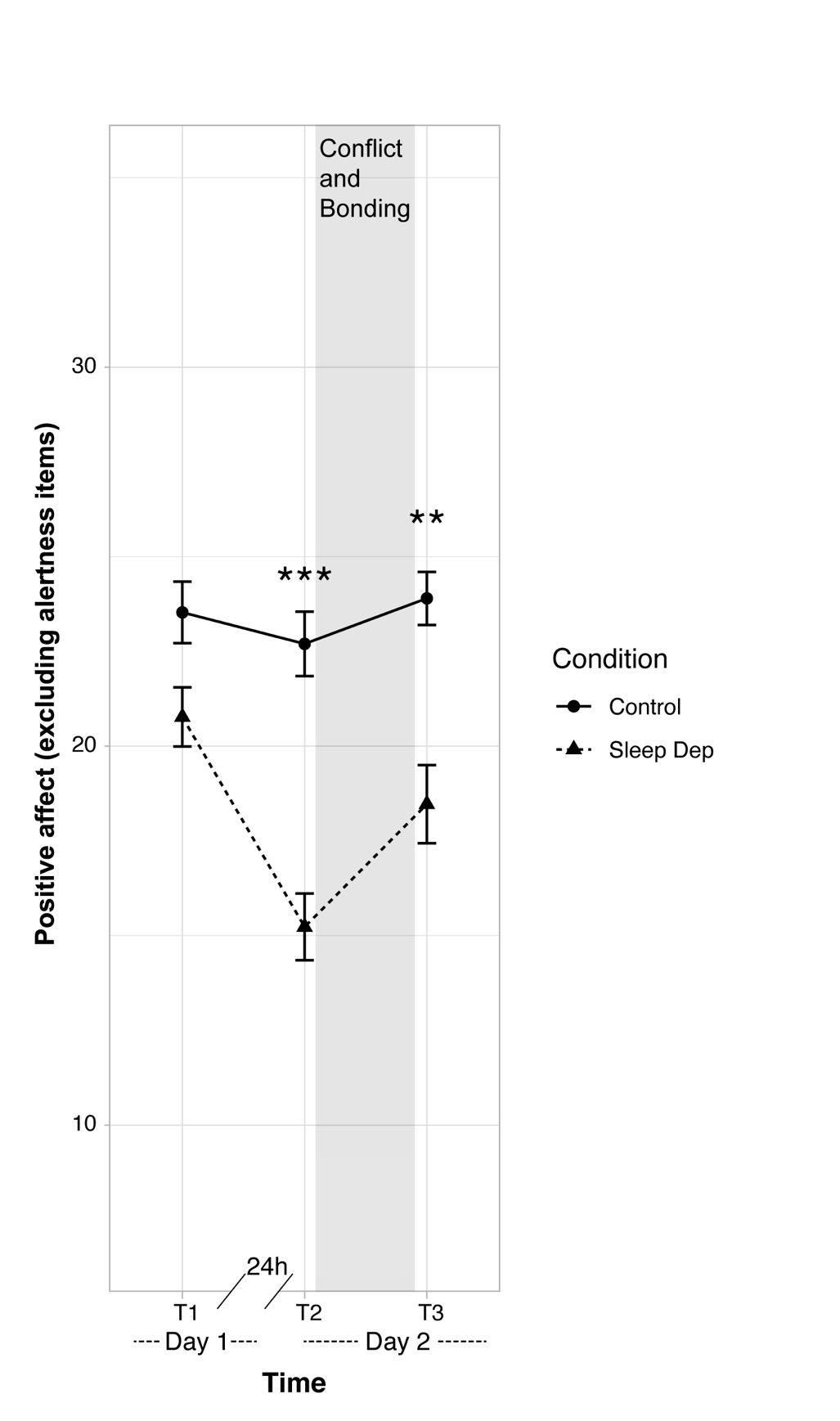


**Supplementary Fig. 2** Mean of positive affect levels (assessed by the Positive Affect Negative Affect Schedule but excluding the three items “active”, “alert”, and “excited”) as a function of condition (Sleep Deprivation, and Control Condition). ****p* < .001, ***p* < .01. Bars represent ± 1 standard errors of the mean. Sleep Dep. = Sleep Deprivation

The 3*2 ANOVA on self-reported negative affect revealed no evidence for an interaction of time × condition, *F*(2, 88) = 0.83, *p* = .44, partial marginal *R^2^* = .007. In addition, no evidence for a main effect of condition, *F*(1, 43) = 0.58, *p* = .45, *β_z_* = 0.21, (95% CI [ -0.32, 0.73]) nor main effect of time, *F*(2, 88) = 0.87, *p* = .42, partial marginal *R^2^* = .015 were found.

*Ratio of positive affect to negative affect*

Previous studies have used a ratio of positive emotions to negative emotions to investigate affect in romantic conflicts (Gordon & Chen, 2014; Gottman, 1994). We obtained the ratio of positive to negative affect using the scores of the two subscales of the PANAS by dividing the sum of positive affect by the sum of negative affect. MLMs indicated that optimal random effects structure was the one including a random subject intercept:

lmer(ratio~condition*time+relationship satisfaction+(1|id subject))

We then conducted a 3*2 ANOVA with time as within-subject factor (T1, T2, T3) and a between-subject factor condition (control vs sleep deprivation). It yielded a significant main effect of condition, *F*(1, 43) = 10.97, *p* = .002, *β_z_* = -0.77, (95% CI [ -1.24, -0.32]), as well as a significant main effect of time, *F*(2, 88) = 6.67, *p* = .002, partial marginal *R^2^* = .069. Likewise, the 3*2 ANOVA revealed a significant interaction effect (time × condition), *F*(2, 88) = 4.48, *p* = .01, partial marginal *R^2^* = .027. Similar to positive and negative analyses, we then conducted contrasts to explore differences between conditions at specific time points. Supplementary Fig. 3 presents the mean of ratio at T1, T2, and T3 for each condition. More precisely, no differences between the conditions were found at T1 (baseline), *t*(85.11) = 1.34, *p* = .18, *β_z_* = -0.38 (95% CI [ -0.94, 0.18]). Sleep-deprived couples showed a lower ratio of positive to negative affect than rested couples at T2 (after the sleep deprivation night), *t*(85.11) = 4.29, *p* < .001, *β_z_* = -1.23 (95% CI [ -1.79, -0.66]), and T3 (after the conflict and bonding discussion), *t*(85.11) = 2.52, *p* = .01, *β_z_* = -0.72. (95% CI [ -1.28, -0.16]). This latter result (T3, after the conflict and bonding discussion) is in line with previous research showing that participants reporting poor sleep also had a lower ratio of positive to negative emotions compared to well-rested participants (Gordon & Chen, 2014).


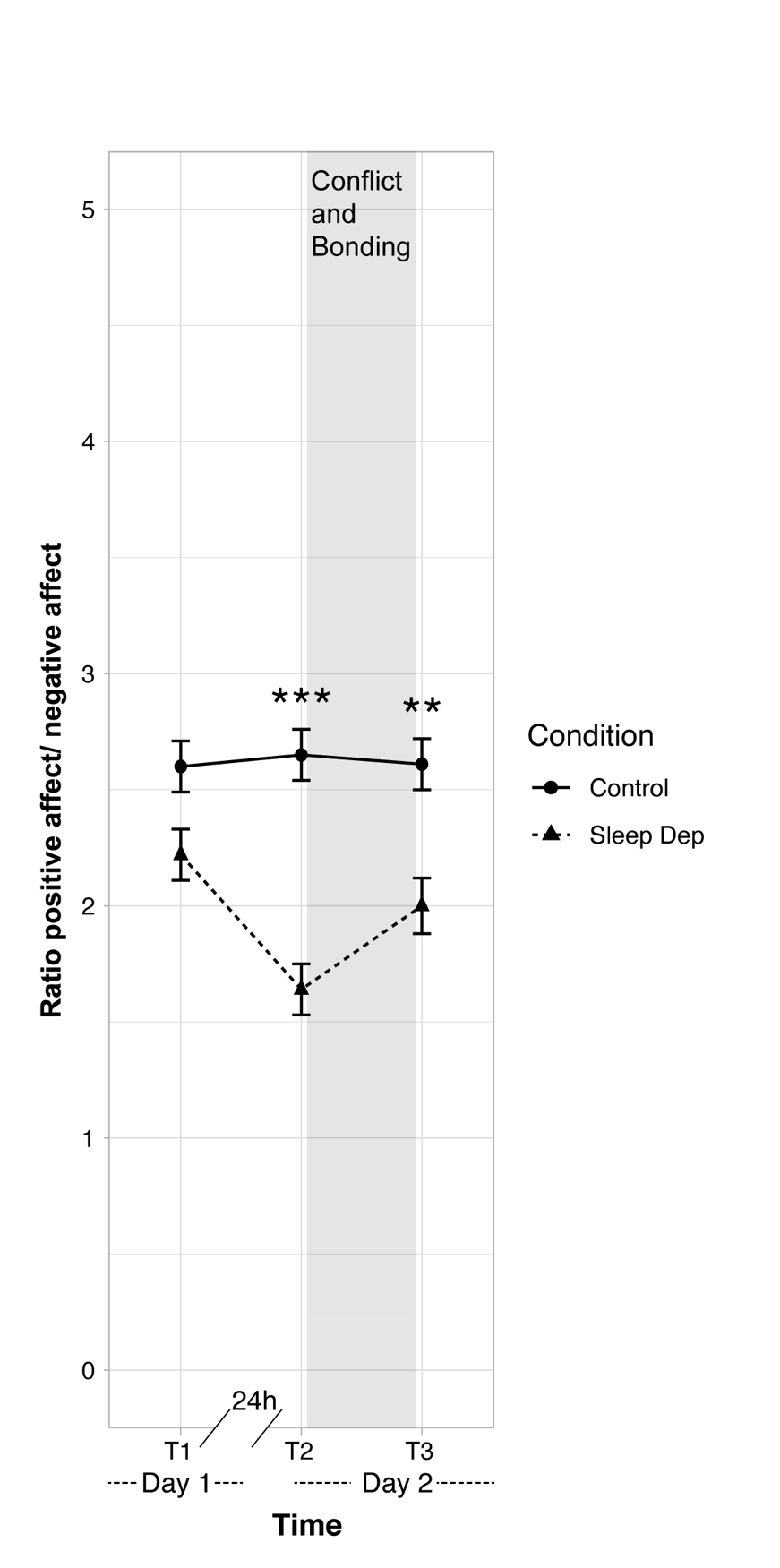


**Supplementary Fig. 3** Mean of ratio of positive affect to negative affect (calculated with PANAS scores) as a function of condition (Sleep Deprivation, and Control Condition). ****p* <.001, ***p* <.01. bars represent ± 1 standard errors of the mean. Sleep Dep. = Sleep Deprivation

*Sleep deprivation effect on conflict-related measures*

Regarding the satisfaction about the agreement and about the content of the conflict discussion, we used MLM analyses to test whether sleep-deprived couples had lower scores on satisfaction about the agreement and satisfaction about the content of the conflict than couples who slept at home. The model testing these two hypotheses included relationship satisfaction scores as a covariate and a random dyad intercept (no repeated measures were done at the level of the subjects). This analysis revealed that sleep-deprived couples and couples who slept at home did not differ related to the satisfaction about the conflict’s content, nor the satisfaction about their agreement, all *p*_s_ ≥ .21. Finally, an MLM analysis was calculated to measure whether sleep-deprived participants and participants who slept at home differed in their ratings related to the severity of the conflict. In addition to the covariate (relationship satisfaction scores), the model selected based on the AIC had a random subject and dyad intercepts. The planned contrast is reported in the main manuscript.

**References**

Hendrick, S. S. (1988). A generic measure of relationship satisfaction. *Marriage and the Family*, *50*(1), 93–98.

Saramago, M., Lemétayer, F., & Gana, K. (2021). Adaptation et validation de la version française de l’échelle

d’évaluation de la relation. Psychologie Française. https://doi.org/10.1016/j.psfr.2020.09.004

Buysse, D. J., Reynolds, C. F., Monk, T. H., Berman, S. R., & Kupfer, D. J. (1989). Pittsburgh Sleep Quality Index ( PSQI ). *Psychiatry Research*, *28*(2), 193–213. https://doi.org/10.1007/978-1-4419-9893-4

Gordon, A. M., & Chen, S. (2014). The Role of Sleep in Interpersonal Conflict: Do Sleepless Nights Mean Worse Fights? *Social Psychological and Personality Science*, *5*(2), 168–175. https://doi.org/10.1177/1948550613488952

Gottman, J. M. (1994). *What predicts divorce?The relationship between marital processes and marital outcomes.* Hillsdale,NJ: Lawrence Erlbaum.

Johns, M. W. (1993). Daytime Sleepiness, Snoring, and Obstructive Sleep Apnea. *Chest*, *103*(1), 30–36. https://doi.org/10.1378/chest.103.1.30

Shahid, A., Wilkinson, K., Marcu, S., & Shapiro, C. M. (2011). Karolinska Sleepiness Scale (KSS). In *STOP, THAT and One Hundred Other Sleep Scales* (Springer N, pp. 209–210). https://doi.org/10.1007/978-1-4419-9893-4_47
